# Supplementary material for: Segmentation of glioblastomas in early post-operative multi-modal MRI with deep neural networks
Source: Sci Rep. 2023 Nov 2;13:18897. doi: 10.1038/s41598-023-45456-x (PMC10622432; doi:10.1038/s41598-023-45456-x)
Supplement: Supplementary file 1 — Supplementary Information. [file 41598_2023_45456_MOESM1_ESM.pdf]

Supplementary Material for Segmentation of glioblastomas in early post-operative multi-modal MRI with deep neural networks

1 Data

Information about scanners, field strengths and acquisition protocols are summarized in Table S1. Some patients are reported as missing, as the DICOM folders were no longer available at the time of submission. For each sequence type, the number and ratio of scans acquired in 3D are reported, and the remaining scans are either acquired in 2D, or the information is missing.

The resolution and spacing of the scans are reported in Table S2, as [min;max] range and mean. The resolution is reported in number of voxels, and the voxel spacing is reported in mm.

Table S1: Description of the dataset in terms of scanner manufacturer, field strength, and acquisition protocols.

**Manufacturer**	
Philips	190 (19.9%)
Siemens	216 (22.6%)
GE	391 (40.9%)
Toshiba	3 (0.3%)
Missing information	156 (16.3%)
**Field strength**	
1T	4 (0.4%)
1.5T	534 (55.9%)
3T	259 (27.1%)
Missing information	159 (16.6%)
**3D Acquisition**	
Postop T1w-CE	569 (59.5%)
Postop T1w	90 (9.4%)
Postop FLAIR	336 (35.1%)
Preop T1w-CE	728 (76.1%)

Table S2: Range and average scan resolution and spacing, for each sequence type.

**Sequence**	Resolution range (voxels)	Resolution mean (voxels)	Spacing range (mm)	Spacing mean (mm)
Postop T1w-CE	[128;896, 42;896, 17;512]	[430, 461, 180]	[0.26;1.2, 0.26;5.0, 0.49;7.2]	[0.67, 0.64, 1.96]
Postop T1w	[160;896, 176;896, 19;512]	[498, 516, 49]	[0.26;1.1, 0.26;1.0, 0.5;7.8]	[0.52, 0.51, 4.81]
Postop FLAIR	[124;576, 178;576, 18;576]	[327, 325, 133]	[0.39;1.3, 0.39;1.37, 0.43;24.0]	[0.76, 0.81, 3.81]
Preop T1w-CE	[160;896, 86;896, 19;512]	[313, 346, 210]	[0.26;1.25, 0.26;2.0, 0.47;7.0]	[0.85, 0.81, 1.27]

2 Statistical analysis

2.1 Summary

Multiple statistical analyses were conducted to compare the two deep learning architectures AGU-Net and nnU-Net on segmentation of glioblastomas. A significance level of 5% was used. Statistical tests were conducted on both the cross-validation splits and the test set.

For segmentation performance on the test set, Tukey's range tests were performed, comparing pairwise differences in means between the individual input configurations. The same tests were conducted on both the AGU-Net and nnU-Net models separately (see Table S3 and S4).

For comparing the best segmentation model for each architecture, a Mann-Whitney U test was conducted on the test set (see Table S5).

To assess classification performance, confidence intervals were computed for each configuration and architecture combination on both the validation cross-validation splits and the test set (see Table S6 and S7).

For comparison of the best segmentation models for each architecture against the four novice and four expert annotators in the inter-rater study, a Mann-Whitney U test was conducted on the inter-rater test set (see Table S8 and S9). The ground truth segmentation was used as a reference (not seen during training), for the purpose of comparing the models and annotators on a non-biased reference, as opposed to using the consensus agreement annotation, as this is by definition biased toward all of the annotators.

2.2 Justification for selection of statistical tests

Tukey for contrast comparison was selected for comparing the different input configurations for each model, as this testing method includes correction of all p-values directly when doing multiple comparisons, and has no strict assumptions on the distribution of the data.

The Mann-Whitney U test was selected for comparing the best segmentation models for each architecture, as the data was not normally distributed, and this test is non-parametric and without any strict assumptions on the data distribution.

In the case of classification, the balanced accuracy metric can only be calculated on a group level over the test set, and therefore the standard deviation could not be computed, as opposed to the segmentation case where the metric is calculated on a patient level. The only possible method for estimating the standard deviation was therefore by bootstrapping, and the BCa intervals were considered the best and most robust alternative to hypothesis testing.

Although the test set should be considered the gold standard for assessing statistical significance, the test set was small (73 patients) and it was difficult to find anything statistically significant. Confidence intervals were therefore also constructed for the cross-validation folds. The balanced accuracy was assumed normally distributed over the five folds. The mean was calculated with pooled estimates, and the standard deviation was calculated over the mean estimates from each fold, as the standard deviation for each fold was not available. This method leads to a conservative estimate for the interval, as the standard deviation was calculated based on the pooled mean estimates across each fold.

For the inter-rater study, the test set was extremely small with only 20 patients, and only 10 patients with residual tumor that could be used for assessing the segmentation performance. All results were computed on pairs of models and annotators, and correction of the p-values was not possible in this case because of the size of the dataset.

2.3 Segmentation study

2.3.1 nnU-Net

Table S3: Multiple comparisons of all input configurations on the test set using the AGU-Net architecture.

group1	group2	meandiff	p-adj	lower	upper	reject
A	B	0.0681	0.4899	-0.0478	0.184	False
A	C	0.0575	0.6318	-0.0584	0.1735	False
A	D	0.0567	0.6433	-0.0592	0.1726	False
A	E	0.0391	0.8782	-0.0768	0.1551	False
B	C	-0.0105	0.9	-0.1265	0.1054	False
B	D	-0.0114	0.9	-0.1273	0.1045	False
B	E	-0.0289	0.9	-0.1449	0.087	False
C	D	-0.0009	0.9	-0.1168	0.1151	False
C	E	-0.0184	0.9	-0.1343	0.0975	False
D	E	-0.0175	0.9	-0.1335	0.0984	False

2.3.2 AGU-Net

Table S4: Multiple comparisons of all input configurations on the test set using the nnU-Net architecture.

group1	group2	meandiff	p-adj	lower	upper	reject
A	B	0.057	0.8102	-0.0924	0.2065	False
A	C	0.0427	0.9	-0.1067	0.1922	False
A	D	0.0279	0.9	-0.1216	0.1773	False
A	E	0.0317	0.9	-0.1178	0.1812	False
B	C	-0.0143	0.9	-0.1638	0.1352	False
B	D	-0.0292	0.9	-0.1787	0.1203	False
B	E	-0.0253	0.9	-0.1748	0.1241	False
C	D	-0.0149	0.9	-0.1644	0.1346	False
C	E	-0.0111	0.9	-0.1605	0.1384	False
D	E	0.0038	0.9	-0.1456	0.1533	False

2.3.3 nnU-Net vs AGU-Net

Table S5: Test set segmentation performance comparison of the AGU-Net and nnU-Net architectures.

DSC-P			
AGU-Net	nnU-Net	Statistic	p-value
43.76 ± 27.61	59.9 ± 20.49	877	0.0023

2.4 Classification study

2.4.1 Test set

Table S6: Test set classification accuracy and confidence intervals for all input configurations for both the AGU-Net and nnU-Net architectures.

Arch	Config	Mean	CI
nnU-Net	A	0.523	[0.500, 0.619]
nnU-Net	B	0.717	[0.611, 0.824]
nnU-Net	C	0.636	[0.553, 0.750]
nnU-Net	D	0.705	[0.604, 0.812]
nnU-Net	E	0.636	[0.552, 0.741]
AGU-Net	A	0.740	[0.615, 0.843]
AGU-Net	B	0.785	[0.665, 0.881]
AGU-Net	C	0.847	[0.743, 0.917]
AGU-Net	D	0.824	[0.704, 0.898]
AGU-Net	E	0.818	[0.697, 0.903]

2.4.2 Validation set

Table S7: Validation set classification accuracy and confidence intervals for all input configurations for both the AGU-Net and nnU-Net architectures.

Model	Config	Mean	CI
nnU-Net	A	0.512	[0.501, 0.522]
nnU-Net	B	0.588	[0.567, 0.608]
nnU-Net	C	0.527	[0.512, 0.543]
nnU-Net	D	0.575	[0.544, 0.606]
nnU-Net	E	0.531	[0.512, 0.549]
AGU-Net	A	0.739	[0.695, 0.782]
AGU-Net	B	0.761	[0.725, 0.797]
AGU-Net	C	0.766	[0.724, 0.809]
AGU-Net	D	0.779	[0.723, 0.835]
AGU-Net	E	0.791	[0.751, 0.831]

2.5 Inter-rater study

Table S8: Test set segmentation performance comparison of both architectures against each annotators, the average of each group of annotators and the average of all annotators using the **ground truth segmentation** from the dataset as the reference.

Model - config	Annotator	Model Mean ± Std	Annotator Mean ± Std	Statistic	p-value
nnU-Net B	nov1	0.395 ± 0.210	0.355 ± 0.286	45.5	0.381
nnU-Net B	nov2	0.395 ± 0.210	0.139 ± 0.145	18.5	0.009
nnU-Net B	nov3	0.395 ± 0.210	0.160 ± 0.200	20.5	0.014
nnU-Net B	nov4	0.395 ± 0.210	0.257 ± 0.240	35.0	0.135
nnU-Net B	exp1	0.395 ± 0.210	0.291 ± 0.196	38.0	0.192
nnU-Net B	exp2	0.395 ± 0.210	0.330 ± 0.248	43.5	0.324
nnU-Net B	exp3	0.395 ± 0.210	0.359 ± 0.211	49.0	0.485
nnU-Net B	exp4	0.395 ± 0.210	0.339 ± 0.180	44.5	0.353
nnU-Net B	nov-avg	0.395 ± 0.210	0.228 ± 0.170	27.0	0.044
nnU-Net B	exp-avg	0.395 ± 0.210	0.330 ± 0.194	42.5	0.298
nnU-Net B	all-avg	0.395 ± 0.210	0.279 ± 0.175	33.5	0.113
AGU-Net B	nov1	0.372 ± 0.225	0.355 ± 0.286	48.0	0.455
AGU-Net B	nov2	0.372 ± 0.225	0.139 ± 0.145	21.0	0.014
AGU-Net B	nov3	0.372 ± 0.225	0.160 ± 0.200	26.0	0.037
AGU-Net B	nov4	0.372 ± 0.225	0.257 ± 0.240	36.0	0.151
AGU-Net B	exp1	0.372 ± 0.225	0.291 ± 0.196	36.0	0.153
AGU-Net B	exp2	0.372 ± 0.225	0.330 ± 0.248	44.0	0.338
AGU-Net B	exp3	0.372 ± 0.225	0.359 ± 0.211	47.0	0.425
AGU-Net B	exp4	0.372 ± 0.225	0.339 ± 0.180	41.0	0.260
AGU-Net B	nov-avg	0.372 ± 0.225	0.228 ± 0.170	31.0	0.080
AGU-Net B	exp-avg	0.372 ± 0.225	0.330 ± 0.194	40.0	0.236
AGU-Net B	all-avg	0.372 ± 0.225	0.279 ± 0.175	35.0	0.136

Table S9: Test set segmentation performance comparison of both architectures against each annotators, the average of each group of annotators and the average of all annotators, using the **consensus agreement annotation** as the reference.

Model - config	Annotator	Model Mean ± Std	Annotator Mean ± Std	Statistic	p-value
nnU-Net B	nov1	0.447 ± 0.188	0.492 ± 0.180	25.0	0.247
nnU-Net B	nov2	0.447 ± 0.188	0.341 ± 0.218	25.0	0.247
nnU-Net B	nov3	0.447 ± 0.188	0.321 ± 0.259	21.0	0.135
nnU-Net B	nov4	0.447 ± 0.188	0.349 ± 0.295	26.0	0.281
nnU-Net B	exp1	0.447 ± 0.188	0.559 ± 0.196	21.0	0.135
nnU-Net B	exp2	0.447 ± 0.188	0.521 ± 0.126	23.0	0.186
nnU-Net B	exp3	0.447 ± 0.188	0.571 ± 0.240	18.0	0.078
nnU-Net B	exp4	0.447 ± 0.188	0.368 ± 0.159	24.0	0.215
nnU-Net B	nov-avg	0.447 ± 0.188	0.376 ± 0.154	23.0	0.186
nnU-Net B	exp-avg	0.447 ± 0.188	0.505 ± 0.133	24.0	0.215
nnU-Net B	all-avg	0.447 ± 0.188	0.440 ± 0.126	31.0	0.479
AGU-Net B	nov1	0.428 ± 0.211	0.492 ± 0.180	25.0	0.247
AGU-Net B	nov2	0.428 ± 0.211	0.341 ± 0.218	25.0	0.247
AGU-Net B	nov3	0.428 ± 0.211	0.321 ± 0.259	24.5	0.231
AGU-Net B	nov4	0.428 ± 0.211	0.349 ± 0.295	31.5	0.500
AGU-Net B	exp1	0.428 ± 0.211	0.559 ± 0.196	23.0	0.186
AGU-Net B	exp2	0.428 ± 0.211	0.521 ± 0.126	20.0	0.114
AGU-Net B	exp3	0.428 ± 0.211	0.571 ± 0.240	15.5	0.046
AGU-Net B	exp4	0.428 ± 0.211	0.368 ± 0.159	22.0	0.159
AGU-Net B	nov-avg	0.428 ± 0.211	0.376 ± 0.154	26.0	0.282
AGU-Net B	exp-avg	0.428 ± 0.211	0.505 ± 0.133	22.0	0.159
AGU-Net B	all-avg	0.428 ± 0.211	0.440 ± 0.126	27.0	0.318
